# Unraveling the complexity of IgG4-related aortitis and periarteritis: from pathogenesis to clinical practice

**DOI:** 10.3389/fimmu.2025.1625456

**Published:** 2025-07-04

**Authors:** Yan Wang, Feng Tian, Hui Li

**Affiliations:** Department of Gastroenterology, Shengjing Hospital of China Medical University, Shenyang, Liaoning, China

**Keywords:** IgG4-related disease, vascular involvement, retroperitoneal fibrosis, diagnostic criteria, treatment strategies

## Abstract

IgG4-related disease (IgG4-RD) is a chronic fibrotic inflammatory condition characterized by elevated serum IgG4 levels and the infiltration of IgG4-bearing plasma cells in affected organs. It can involve various organs, particularly large vessels. IgG4-related aortitis/periaortitis and periarteritis (IgG4-related PAO/PA) predominantly affect the abdominal aorta and iliac arteries, with a higher prevalence in elderly males. This condition exhibits distinct clinical, histologic, and radiological features compared to IgG4-RD without vascular involvement and other immune-associated vasculitides. IgG4-related PAO/PA diagnosis primarily relies on histopathological findings and imaging studies. Glucocorticoids (GCs) are the mainstay of treatment, often combined with immunosuppressants (IMs), while B- and T-cell-targeted therapies are under investigation. Although most patients respond well to treatment, the disease can be life-threatening due to complications such as myocardial infarction, aortic dissection, and aneurysmal rupture. Therefore, understanding these characteristics is crucial for clinicians to make accurate diagnoses and implement effective treatment strategies. This review provides a comprehensive overview of the current understanding of the pathogenesis, histopathological characteristics, clinical features, diagnosis, treatment, and prognosis of IgG4-related PAO/PA.

## 1 Introduction

IgG4-RD is a systemic autoimmune disorder characterized by the infiltration of IgG4-positive plasma cells into various organs, elevated serum IgG4 levels, and resultant chronic inflammation and fibrosis (1). It was first identified in 2001 among Japanese patients with autoimmune pancreatitis (AIP) (1). Subsequently, Kamisawa et al. proposed the term “IgG4-related autoimmune disease” to describe multi-organ involvement in AIP (2). By 2012, an international consensus standardized the nomenclature as “IgG4-RD” to encompass systemic manifestations (3).

The precise prevalence of IgG4-RD remains unknown. However, recent U.S. epidemiological studies (2015–2019) estimate an incidence of 0.78–1.39 per 100,000 person-years and a point prevalence of 5.3 per 100,000 as of 2019. Mortality rates were 3.42 and 1.46 deaths per 100 person-years in cases versus comparators, respectively (4). The pathogenesis involves a dominant Th2 immune response, driving the production of cytokines (IL-4, IL-5, IL-10, and IL-13) that promote B-cell class-switching to IgG4 production and fibrosis (5, 6). Activated B cells further enhance CD4^+^ T-cell activity, while cytotoxic CD4^+^ T lymphocytes contribute to tissue damage through apoptosis and the release of pro-fibrotic mediators, such as transforming growth factor-beta (TGF-β) (7). IgG4-RD predominantly affects middle-aged to older men and is histologically characterized by lymphoplasmacytic infiltration, storiform fibrosis, and an abundance of IgG4^+^ plasma cells (8–10). It can involve nearly any organ, commonly presenting as AIP, sclerosing cholangitis, sialadenitis, dacryoadenitis, tubulointerstitial nephritis, or retroperitoneal fibrosis (RF) (11). Vascular involvement in IgG4-RD has been increasingly recognized. In 2008, the first case associating inflammatory abdominal aortic aneurysms (AAAs) with IgG4-RD was reported (12). By 2012, IgG4-RD was formally recognized as a cause of aortitis in the revised Chapel Hill Consensus (13).

IgG4-related PAO/PA is a critical subset of IgG4-RD, characterized by inflammation of the aortic wall (“aortitis”), adjacent tissues (“periaortitis”), or medium-sized arteries (“periarteritis”) (3, 14–17). The abdominal aorta, particularly the infra-renal portion, and the iliac arteries are most frequently affected. However, the thoracic aorta and its branches, such as the carotid and coronary arteries, may also be involved (17, 18). Clinically, IgG4-related PAO/PA is frequently misdiagnosed as infectious or autoimmune vasculitis due to its nonspecific symptoms. Complications such as aneurysm formation, dissection, and rupture are life-threatening (19, 20). This review aims to provide a comprehensive overview of the current understanding of IgG4-related PAO/PA, including its epidemiology, pathogenesis, clinical features, diagnosis, treatment, and prognosis.

## 2 Epidemiology of IgG4-related PAO/PA

Epidemiological data on IgG4-related PAO/PA is currently limited. Prevalence estimates are often derived from case series of IgG4-RD or studies on idiopathic RF that later identified vascular involvement as a component of IgG4-RD. A systematic review indicated that 10% to 30% of patients with IgG4-RD exhibit vascular involvement (21).

IgG4-related PAO/PA predominantly affects middle-aged to elderly individuals, typically in their fifth to seventh decades of life, with a higher incidence in males. However, there have also been documented cases in females (21). Most research on IgG4-related PAO/PA has been conducted in Japan, where the disease is more widely recognized, while data from Western countries are limited.

The proportion of IgG4-related PAO/PA among IgG4-RD cases varies by region: it ranges from 6% to 41% in Japan (17, 22–26), 4% to 26% in China (20, 27–29), 10% to 23% in the USA (15, 30, 31), and 6%-36% in other countries (32–36). Vascular involvement is primarily identified through radiological imaging (**Table 1**). Based on computed tomography (CT) imaging features, IgG4-related PAO/PA was diagnosed in 12% to 36% of IgG4-RD cases (17, 22). A study utilizing fluorodeoxyglucose positron emission tomography/computed tomography (FDG-PET/CT) combined with contrast-enhanced CT (CECT) found that 41% of patients with IgG4-RD had vascular involvement (26). Histopathological assessments have reported vascular involvement in IgG4-RD cases ranging from 6% to 23% (15, 23). For instance, Wallace et al. identified aortic involvement in 11% of patients with biopsy-confirmed IgG4-RD (31). Furthermore, various diagnostic criteria, including the comprehensive diagnostic criteria (CDC), the 2020 revised comprehensive diagnostic criteria (RCD), and the 2019 American College of Rheumatology/European League Against Rheumatism classification criteria (AECC), have indicated that 11% to 26% of IgG4-RD patients exhibit lesions in the retroperitoneum or periaorta (24, 27, 28, 35). The epidemiological characteristics of IgG4-related PAO/PA are detailed in **Table 2**.

**Table 1:** A summary of vascular involvement in IgG4-RD.

Author (year)	Country	Diagnostic criteria of IgG4-RD	No. of IgG4 RD	Diagnosis of Vascular involvement	Vascular Involvement, n (%)	Ref.
Zen et al. (2010)	Japan	Pathology	114	Radiology	Aorta/artery, 7 (6%)	(23)
Ebbo et al. (2012)	France	Organ involvement, serum, and pathology	25	Pathology, radiology	Abdominal aorta, 3 (12%); thoracic aorta, 6 (24%)	(32)
Mizushima et al. (2014)	Japan	CDC/Organ-specific diagnostic criteria	333	Radiology	Periaortiti/periarteritis, 40 (12%)	(22)
Chen et al. (2014)	China	CDC	28	Radiology	Aorta, 1 (4%)	(29)
Wallace et al. (2015)	USA	CDC	125	Radiology	Aorta, 14 (11%)	(31)
Lin et al. (2015)	China	CDC	118	Radiology	Periaortitis/RF, 31 (26%)	(28)
Perugino et al. (2016)	USA	Pathology	160	Pathology, radiology	Aortitis/periaortitis, 36 (23%)	(15)
Yamada et al. (2017)	Japan	CDC/Organ-specific diagnostic criteria	334	Pathology, radiology	RF/periaorta, 83 (25%)	(24)
Ozawa et al. (2017)	Japan	CDC	179	Radiology	Periaortitis/periarteritis, 65 (36%)	(17)
Yabusaki et al. (2017)	Japan	CDC	37	Pathology, radiology	Aortitis, 15 (41%)	(26)
Wallace et al. (2019)	USA	AECC	493	Radiology	Aorta, 51 (10%)	(30)
Lanzillotta et al. (2020)	Italy	Pathology/CDC	131	Radiology	Aorta, 12 (9%)	(33)
Peng et al. (2020)	China	CDC	587	Radiology	Periaortitis/periarteritis, 89 (15%)	(20)
Fernández-Codina et al. (2021)	Spanish	JCC and IPC	105	N	Aorta, 12 (13%); arteries, 4 (4%)	(34)
Ashihara et al. (2022)	Japan	CDC	104	Radiology	Periaortitis/periarteritis, 38 (37%)	(25)
Vikse et al. (2023)	Norwegian	CDC, RCD, and AECC	79	N	RF/aorta, 18 (22.8%)	(35)
Martín-Nares et al. (2024)	Latin America*	CDC/Pathology/RCD	180	Radiology	Thoracic aorta, 4 (2.2%); abdominal aorta, 6 (3.3%)	(36)
An et al. (2024)	China	AECC	605	Radiology	Aorta, 14 (2%); RF/aortitis, 64 (11%)	(27)

IgG4-RD, IgG4-related disease; CDC, comprehensive diagnostic criteria for IgG4-RD; RCD, the 2020 revised comprehensive diagnostic criteria for IgG4-RD; 2019 AECC, the 2019 American College of Rheumatology/European League Against Rheumatism classification criteria; JCC, Japanese comprehensive criteria; IPC, International pathology consensus; RF, retroperitoneal fibrosis; N, not mentioned. Note: *Argentina, Chile, Mexico, Peru, and Uruguay.

**Table 2:** Epidemiological characteristics of IgG4-related PAO/PA.

Author (year)	Country	No. Total	No. Definite	No. Probable	No. Possible	Age, years	Male, n%	Most affected, n (%)	Aneurysmal change	Extravascular involvement	Serum IgG4 level, mg/dL	Ref.
Kasashima et al. (2009)	Japan	13	NS	NS	NS	70 (median)	11 (85%)	Iliac arteries,1 (8%)	13 (100%)	NS	274 (mean)	(56)
Ioue et al. (2011)	Japan	17	NS	NS	NS	65 (mean)	17 (94%)	Iliac arteries,13 (76%)	2 (12%)	12 (71%)	672 (mean)	(18)
Mizushima et al. (2014)	Japan	40	25	3	12	66 (mean)	37 (93%)	Abdominal aortas, 33 (83%)	3 (8%)	36 (90%)	815 (mean)	(22)
Ebe et al. (2015)	Japan	7	6	NS	1	67 (mean)	6 (86%)	Abdominal aorta,6 (86%)	None	6 (86%)	933 (mean)	(74)
Castelein et al. (2015)	Belgium	9	NS	NS	NS	61 (median)	9 (100%)	Infrarenal aorta, 6 (67%)	NS	8 (89%)	511 (mean)	(73)
Perugino et al. (2016)	USA	36	NS	NS	NS	55 (mean)	28 (78%)	Thoracic aortitis, 8 (22%)	11 (31%)	34 (94%)	99 (median)	(15)
Ozawa et al. (2017)	Japan	65	NS	NS	NS	69 (median)	53 (82%)	Iliac arteries, 29 (45%)	8 (19%)	65 (100%)	511 (median)	(17)
Kim et al. (2017)	South Korea	10	0	1	9	69 (median)	6 (60%)	Abdominal aorta, 7 (70%)	3 (30%)	7 (70%)	193 (median)	(16)
Yabusaki et al. (2017)	Japan	15	15	0	0	70 (median)	12 (80%)	Infrarenal abdominal aorta,12 (80%)	10 (67%)	15 (100%)	768 (median)	(26)
Kasashima et al. (2018)	Japan	32 (IgG4-AA:24; IgG4-PA:8)	NS	NS	NS	IgG4-AA:81 (median); IgG4-PA:77 (median)	IgG4-AA:19 (79%); IgG4-PA:7 (88%)	IgG4-AA: abdominal aorta,12 (50%) IgG4-PA: abdominal aorta,7 (88%)	24 (75%)	NS	IgG4-AA:254 (median); IgG4-PA:282 (median)	(86)
Qi et al. (2019)	China	21	18	0	3	52 (mean)	15 (71%)	Thoracic aorta, 14 (67%)	10 (48%)	19 (90%)	433 (median)	(75)
Mizushima et al. (2019)	Japan	99	24	5	76	67 (mean)	84 (84.8%)	Abdominal aorta, 67 (68%)	NS	72 (73%)	551 (median)	(72)
Peng et al. (2020)	China	89	35	1	53	58 (mean)	76 (85%)	Abdominal aorta, (74, 83%)	9 (10%)	65 (73%)	4240 (median)	(20)
Ashihara et al. (2022)	Japan	38	NS	NS	NS	59 (median)	31 (82%)	NS			673 (median)	(25)

IgG4-related PAO/PA, IgG4-related aortitis/periaortitis and periarteritis; IgG4-AA, IgG4-related aortic aneurysm; IgG4-PA, IgG4-related periaortitis; NS, not specified.

Several factors may contribute to the variability in IgG4-related PAO/PA prevalence rates. One significant factor is the under-recognition of vascular involvement in IgG4-RD, which can lead to missed diagnoses. Additionally, differentiating IgG4-related PAO/PA from other types of vasculitis based on radiological findings presents a challenge for clinicians. The variability in diagnostic criteria further complicates the accurate identification of this condition. Addressing these issues is essential for enhancing the understanding and recognition of IgG4-related PAO/PA, ultimately leading to improved patient outcomes (37).

## 3 Potential mechanisms of IgG4-related PAO/PA

The precise pathogenesis of IgG4-related PAO/PA remains incompletely understood. However, emerging evidence suggests that the disease arises from a complex interplay of immune dysregulation, cytokine imbalances, genetic predisposition, and potential environmental triggers. This section explores the multifactorial mechanisms underlying IgG4-related PAO/PA, focusing on immune-mediated processes, cytokine networks, and genetic associations.

### 3.1 Immune dysregulation and cytokine networks

IgG4-RD is characterized by a dysregulated immune response, primarily involving the interplay between Th cell subsets and B cells. In IgG4-related PAO/PA, the balance between T helper (Th) 1 cells and Th2 cells is disrupted, leading to a predominant Th2 response. This shift produces excessive cytokine production, such as IL-4, IL-5, IL-10, and IL-13, which drive B cells to produce IgG4 and promote fibrosis (38–42).

Dysregulation of T-follicular helper cells and their interactions with other immune cells may contribute to the pathogenesis of IgG4-RD across multiple organs, including the aorta and its surrounding tissues (43, 44). A recent study by Kasashima et al. provided a comprehensive analysis of whole-slide immunohistochemical images from surgical specimens of patients with different types of AAAs, including those related to IgG4, non-IgG4 inflammatory AAAs, atherosclerotic AAAs, and Takayasu arteritis (TA). The study revealed that morphological changes in the number, size, and shape of adventitial tertiary lymphoid organs (TLOs) in IgG4-related AAAs, along with an increased presence of T follicular regulatory (Tfr) cells, are closely associated with the disease activity of IgG4-related disorders. Arterial/aortic TLOs and Tfr cells may also be crucial in developing and progressing IgG4-related AAAs (45).

The inflammatory and fibrotic processes of IgG4-RD are primarily regulated by cytokines secreted by Th2 cells, including IL-4, IL-5, and IL-13, along with Treg that produce IL-10 and TGF-β. Among these, IL-4 is crucial in prompting B cells to switch to producing IgG4, while IL-13 intensifies tissue fibrosis by activating fibroblasts and encouraging collagen deposition (46). TGF-β is another significant fibrogenic cytokine found in elevated levels in IgG4-related tubulointerstitial nephritis, which drives collagen production and matrix remodeling, leading to ongoing fibrosis (47). IL-10, known for its anti-inflammatory properties, aids in the differentiation of B cells into plasma cells, which results in increased IgG4 production. In the context of IgG4-related aortic aneurysm, there is a notable local increase in IL-10 and IL-13 within the aortic adventitia, indicating the involvement of Th2 and Treg immune responses (48). Recent studies have also highlighted the involvement of IL-6, a pro-inflammatory cytokine, in IgG4-related PAO/PA. Elevated levels of IL-6 have been observed in the aortic adventitia of patients with IgG4-aortic aneurysms, correlating with disease activity and serum IgG4 levels (48, 49). IL-6 promotes B cell differentiation and the production of IgG4 while also contributing to fibrosis through the activation of fibroblasts and collagen deposition (50–53). Targeting IL-6 with inhibitors has shown promise in reducing inflammation and fibrosis in preclinical models and clinical trials (48).

### 3.2 Allergic and infectious factors

Allergic mechanisms may also contribute to the development of IgG4-related PAO/PA. Elevated serum IgE levels and peripheral eosinophilia are observed in 40% of patients with IgG4-RD, suggesting a potential role for type 2 immune responses in disease pathogenesis (54–57). Studies have reported that 36% to 38% of patients with IgG4-related PAO/PA have a history of allergies, compared to higher rates in patients with non-vascular IgG4-RD (17, 58). According to a study by Peng et al., 28% of patients with periarteritis had a history of allergies. They further compared the prevalence of allergic diseases in IgG4-related PAO/PA patients with and without extraglandular involvement. They found that allergic diseases were more prevalent in patients with lacrimal and/or salivary gland lesions than in those without (54% versus 16%) (20). These findings indicate that allergic conditions may influence the clinical phenotype and severity of IgG4-RD. In addition, infectious agents have been proposed as potential triggers for IgG4-related PAO/PA. Although direct evidence linking infections to disease onset is limited, IgG4-positive plasma cells in infectious aortitis suggest that infections may contribute to immune dysregulation and subsequent development of IgG4-RD (59, 60).

### 3.3 Genetic predisposition

**Figure 1:**
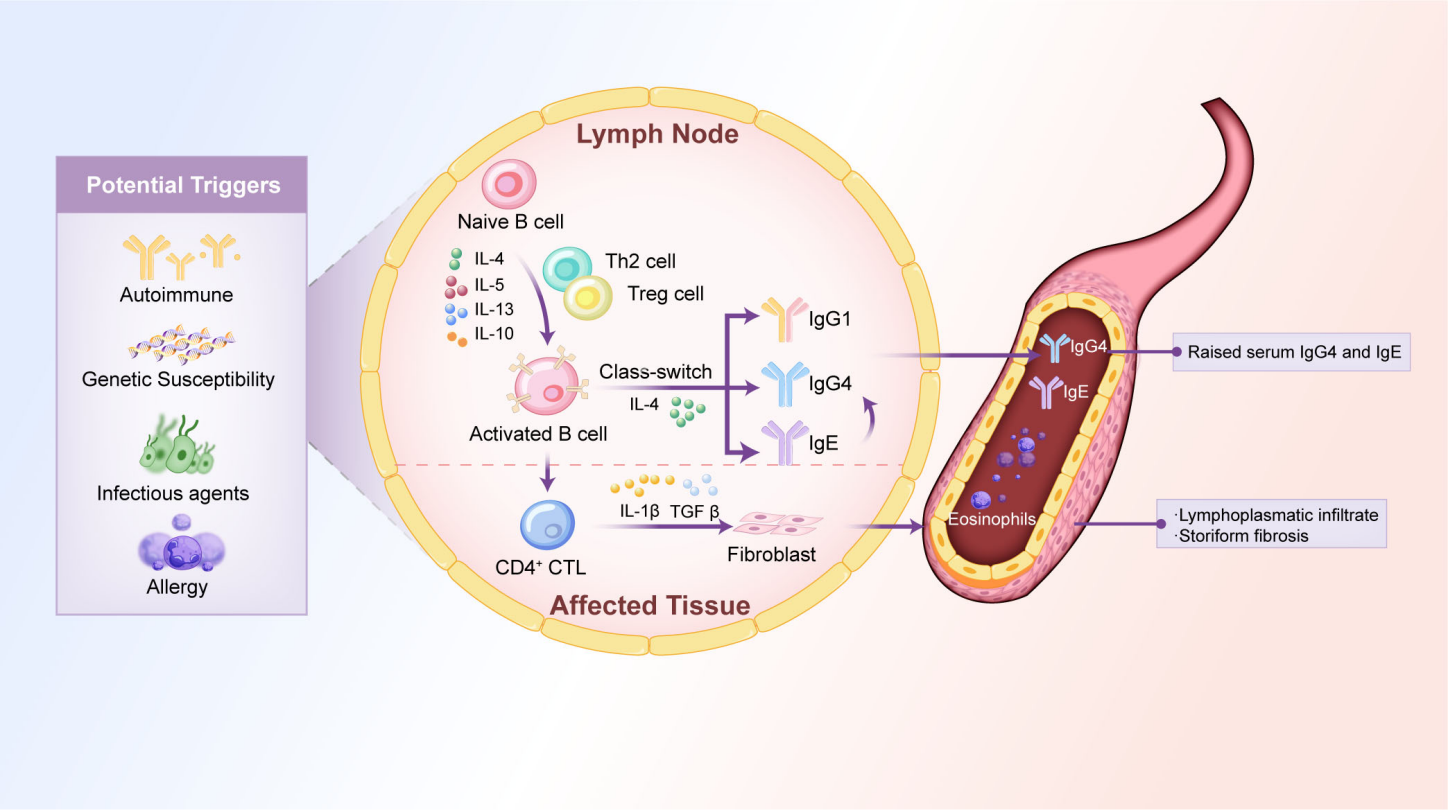
Potential mechanisms of IgG4-related PAO/PA. The pathogenesis of IgG4-related PAO/PA is associated with cytokines, immune dysregulation, genetic predisposition, infectious agents, and allergic reactions. These factors disrupt the balance between Th1 and Th2 cells, leading to the overproduction of Th2 cell-derived cytokines (e.g., IL-4, IL-5, IL-13) and Treg cell-derived cytokines (e.g., IL-10, TGFβ). These cytokines activate naïve B cells, promoting IgG4 class-switching, which leads to elevated levels of serum IgG4 and IgE and tissue damage characterized by lymphoplasmacytic infiltrate and storiform fibrosis. Th, T helper; IL, interleukin; Treg cell, regulatory T-cell; TGFβ, transforming growth factor β; CD4^+^CTL, CD4^+^ cytotoxic T lymphocyte.

Genetic factors are increasingly recognized as essential contributors to IgG4-related PAO/PA pathogenesis. Genome-wide association studies have identified specific *HLA* class II alleles, particularly *HLA-DRB1* and *HLA-DQB1*, as risk factors for IgG4-RD (61). These alleles are involved in antigen presentation to T cells and may predispose individuals to an immune response favoring IgG4 production and chronic inflammation. Other genetic variants associated with IgG4-related PAO/PA include polymorphisms in the *IL-1 receptor type 1* gene, which encodes the IL-1 receptor and is involved in inflammatory signaling (62). However, further studies are needed to elucidate the precise genetic mechanisms underlying IgG4-related PAO/PA. The potential pathogenesis of IgG4-related PAO/PA is illustrated in **Figure 1**.

## 4 Histologic features of IgG4-related PAO/PA

Histopathological examination is crucial for diagnosing IgG4-related PAO/PA and differentiating it from other vasculitis and inflammatory conditions. The histologic features of IgG4-related PAO/PA are characterized by chronic inflammation and fibrosis, primarily involving the adventitia of the aorta and large arteries (58). These features are distinct from those observed in other large-vessel vasculitides, such as giant cell arteritis (GCA) and TA, which typically spare the adventitia and primarily involve the intima and media layers (37).

### 4.1 Lymphoplasmacytic infiltration

The hallmark histologic feature of IgG4-related PAO/PA is dense lymphoplasmacytic infiltration, with a significant presence of IgG4-positive plasma cells. These infiltrates are predominantly located in the adventitia of the aorta or other affected arteries, leading to the thickening of the vessel wall (58). Diagnostic criteria based on biopsy specimens specify >10 IgG4-positive plasma cells per high-power field (HPF) and an IgG4/IgG ratio >40%; for surgical specimens, the thresholds are more stringent: >30 IgG4-positive plasma cells per HPF and an IgG4/IgG ratio >40% (37, 63). It is important to note that IgG4-positive plasma cell infiltration is not exclusive to IgG4-RD. However, the presence of IgG4-positive plasma cells, in conjunction with other histologic features such as storiform fibrosis and obliterative phlebitis, strongly supports the diagnosis of IgG4-related PAO/PA.

### 4.2 Storiform fibrosis

Storiform fibrosis is a key histologic feature of IgG4-RD and is frequently observed in IgG4-related PAO/PA. This pattern of fibrosis is characterized by whorled or storiform arrangements of collagen fibers in the adventitia, resulting in the thickening and rigidity of the vessel wall (12). Storiform fibrosis indicates chronic inflammation and is associated with disease progression, particularly in cases where it leads to complications such as aneurysm formation or vascular obstruction (12).

### 4.3 Obliterative phlebitis

Obliterative phlebitis, another characteristic histologic feature of IgG4-related PAO/PA, is defined by the occlusion of veins due to dense inflammatory infiltrates. This leads to the destruction or narrowing of venous structures. This feature helps distinguish IgG4-related PAO/PA from other types of vasculitis, which typically do not exhibit such marked venous involvement (64).

### 4.4 Eosinophil infiltration

Eosinophil infiltration is frequently observed in IgG4-related PAO/PA, with studies reporting its presence in up to 85% of cases (58, 65). Eosinophils contribute to the inflammatory milieu and may play a role in tissue remodeling and fibrosis by releasing cytokines such as IL-5 and TGF-β. While eosinophil infiltration is not specific to IgG4-RD, its presence in conjunction with other histologic features supports the diagnosis (58).

### 4.5 Diagnostic limitations

Histopathological evaluation has several limitations. Firstly, IgG4-positive plasma cells lack pathognomonic specificity for IgG4-RD and can be prominent in malignancies, infections, other vasculitides, and even chronic aortic dissection, leading to potential false positives (59, 60, 66–71). Secondly, key features like storiform fibrosis or obliterative phlebitis are often missed in small biopsy samples and are more reliably identified in larger surgical specimens. Thirdly, substantial histological overlap exists with conditions like idiopathic RF and atherosclerosis, as features such as lymphoplasmacytic infiltration are not unique to IgG4-RD. Therefore, histopathological findings must always be interpreted in the context of the overall clinical presentation, serological markers, and radiological imaging to achieve an accurate diagnosis.

## 5 Clinical features of IgG4-related PAO/PA

### 5.1 Nonspecific symptoms

Patients with IgG4-related PAO/PA often present with nonspecific systemic symptoms and vascular-associated symptoms. The most common symptom is abdominal or back pain, typically localized to the lower abdomen or lumbar region (20, 72). Other frequent complaints include fatigue, weight loss, and malaise, which may appear early in the disease course and are easily overlooked, leading to delayed diagnosis (12). Notably, up to 30% of patients with IgG4-RD are asymptomatic, which may be higher in those with IgG4-related PAO/PA (72).

Extravascular organ involvement is common in IgG4-related PAO/PA, affecting 71% to 100% of patients (15–18, 20, 22, 26, 72–75). The most frequently involved non-vascular organs are the pancreas, lymph nodes, and salivary glands (72). However, up to 30% of patients with IgG4-related periaortitis may present without involvement of other organs, categorizing them as having isolated aortitis (16). Regular clinical follow-up is essential for patients with isolated vascular involvement, as the disease can progress to affect other organs. Early detection is crucial for effective management.

### 5.2 Vascular-associated symptoms

IgG4-related PAO/PA is characterized by a wide range of vascular lesions, with symptoms closely linked to the specific location and extent of arterial involvement. The most frequently affected vessels are the abdominal aorta, iliac arteries, and thoracic aorta (76). Abdominal aortitis is a common form of IgG4-related PAO/PA, often presenting as periaortitis involving the abdominal aorta and concurrent RF. Patients typically report abdominal pain, particularly in the lower abdomen or back (26, 77). Thoracic aortic lesions may cause chest pain, shortness of breath, or signs of mediastinal compression and can lead to serious complications such as aneurysm formation or dissection (15, 78, 79).

IgG4-related PAO/PA can also involve the major branches of the aorta, affecting the infra-renal portion of the abdominal aorta and extending to the iliac arteries, characterized by stenosis or aneurysmal changes in the affected vessels. Systematic reviews have indicated that the infra-renal abdominal aorta and iliac arteries are the most commonly involved sites, with involvement rates ranging from 52% to 100% in cases of IgG4-related PAO/PA (21). When the renal artery is affected, it can lead to ischemic nephropathy or hypertension, while involvement of the mesenteric artery may result in abdominal angina or bowel ischemia. Patients with iliac or femoral artery lesions may experience claudication, which manifests as leg pain during walking or exertion due to reduced blood flow (21).

Several factors may explain the predilection of IgG4-related PAO/PA for the infra-renal aorta, including distinct histological and pathological traits influenced by blood flow, arteriosclerosis, and vascular changes. The progression of arteriosclerosis below the renal arteries is prevalent and may contribute to developing aortic aneurysms (80). Castelein et al. noted no significant difference in the distribution of periaortic lesions between IgG4-related and idiopathic periaortitis groups, but the former exhibited higher calcium content in the aortic wall. They proposed that atherosclerotic plaques might contribute to IgG4-related periaortitis and suggested that vessel lesions could be associated with the surrounding adventitia (73). Additionally, Ozawa et al. found that patients with IgG4-related PAO/PA had a higher incidence of kidney and urinary tract involvement compared to those without vascular involvement, indicating that inflammation in these regions might affect the localization of the disease (17).

In certain instances, IgG4-related PAO/PA can manifest with multisegmental involvement of the thoracic and abdominal aorta and their branches. However, 20% of patients exhibit vascular involvement at a single site (26). The diffuse form of the disease is linked to a more aggressive clinical course and extensive systemic involvement. Patients with diffuse disease may show symptoms affecting multiple organ systems and face an increased risk of complications, such as aneurysms or dissections occurring in various segments of the aorta (19).

### 5.3 IgG4-related RF

IgG4-related RF is a rare condition in the spectrum of IgG4-RD, affecting approximately 3% to 19% of patients diagnosed with IgG4-RD. It is characterized by chronic inflammation and fibrosis in the retroperitoneal space, often involving the adventitia of the abdominal aorta, iliac arteries, and adjacent structures (81, 82). Thus, the vascular involvement in IgG4-related RF may also be considered secondary vasculitis (15). However, there is considerable overlap between primary vascular conditions, such as aortitis, periaortitis, and periarteritis, and vascular lesions secondary to IgG4-related RF (3, 16). Recognizing that these manifestations can co-occur in the same patient is crucial (15).

IgG4-related RF often affects the abdominal aorta, starting in the infra-renal region and extending caudally to the iliac arteries (81). It can also result in medialization of the ureters and hydronephrosis, which frequently leads to permanent renal injury due to post-obstructive nephropathy (82). Clinical distinctions exist between primary and secondary vascular diseases within IgG4-RDs. Patients with primary IgG4-related vasculitis exhibit higher levels of inflammatory markers, including total serum IgG, IgG1, IgG4, and C-reactive protein (CRP) (15). These patients are significantly more likely to present signs or symptoms directly related to vascular involvement. In contrast, only 13% of patients with secondary vasculopathy reportedly present with vascular-associated signs or symptoms (18, 22). The key characteristics of secondary IgG4-related vasculopathy include perivascular soft tissue enhancement and thickening and FDG avidity on PET scans, yet inflammation within the vessel wall itself is typically minimal. Moreover, primary vasculitis is linked to a higher risk of aneurysm formation and, occasionally, dissection or perforation. In contrast, secondary IgG4-related vasculopathy is associated with a lower risk for aneurysm formation but is more likely to lead to arterial stenosis. Recognizing these distinctions is vital for precise diagnosis and tailored treatment plans for patients with IgG4-RD (15).

### 5.4 Complications of IgG4-related PAO/PA

The progression of IgG4-related PAO/PA may lead to serious, life-threatening vascular complications due to chronic inflammation and fibrosis in the arterial walls. This compromise in vascular integrity may result in dilation and aneurysm formation, especially in the abdominal aorta. Research has indicated that inflammatory AAAs are the most prevalent lesions associated with IgG4-RD, accounting for about 5% of all surgical AAAs and 50% of all inflammatory AAAs. It is estimated that between 8% and 100% of patients with IgG4-related PAO/PA may be affected by this condition (21, 79, 83–85). According to a study by Qi et al., aneurysms are primarily found in the ventral aorta and aortic arch, with dilation also occurring in the iliac arteries (75). Common symptoms of these aneurysms include low-grade fever, abdominal or lumbar pain, and hydronephrosis (86). Many patients with AAAs may remain asymptomatic until the aneurysm reaches a critical size or ruptures. Thoracic aortic aneurysms can manifest with chest pain or symptoms indicative of compression on adjacent structures, such as the airways or esophagus (18).

**Figure 2:**
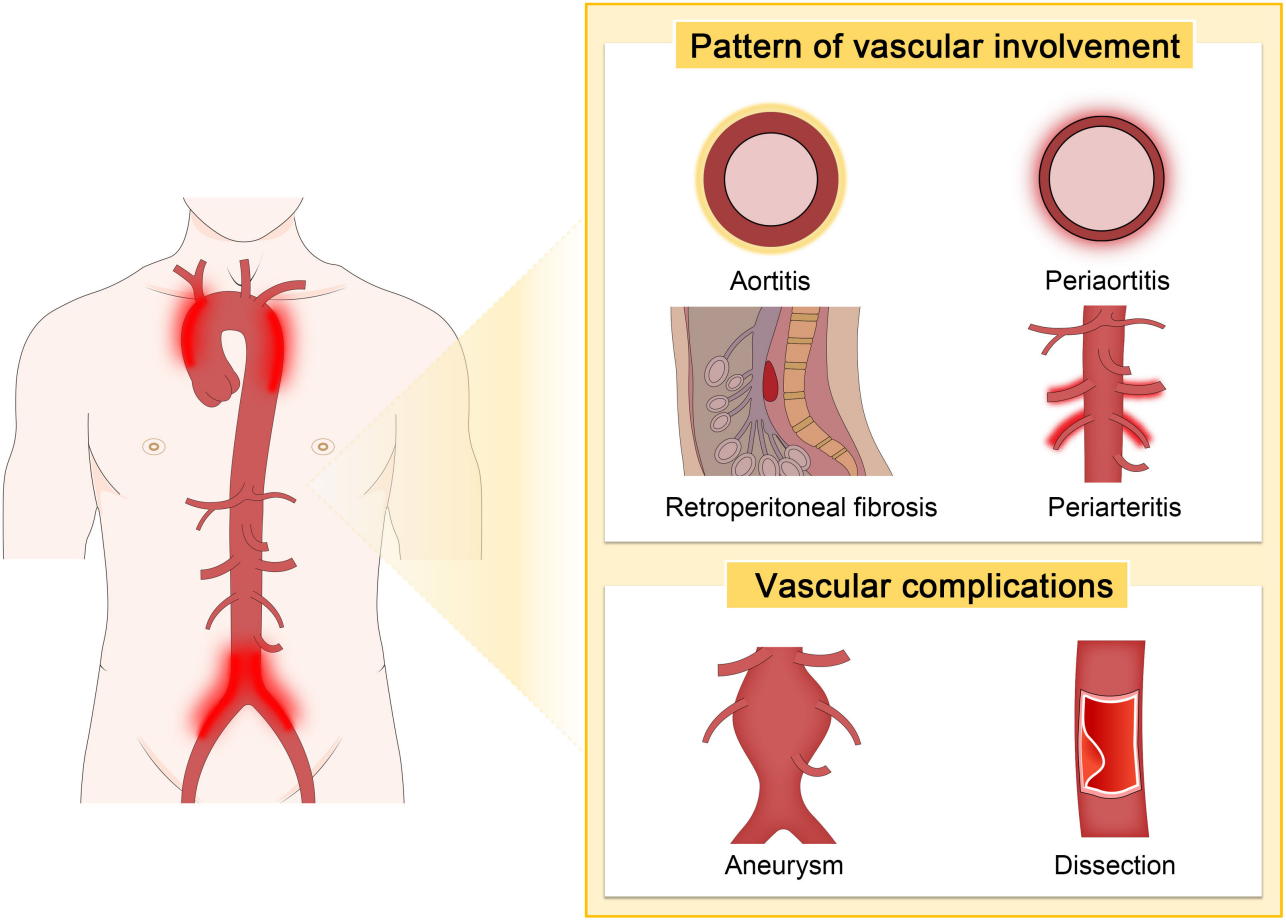
The vascular patterns and common complications of IgG4-related PAO/PA.

Kasashima et al. reported that aneurysmal rupture was less common in IgG4-related inflammatory AAAs compared to non-IgG4 cases (30% vs. 0%) (58). In contrast, Palazzo et al. noted an alarmingly high rate of aneurysmal rupture, reporting 42% of cases of IgG4-related aortitis (87). A less common but more severe complication associated with IgG4-RD is aortic dissection. Although rare, several cases of thoracic aortic aneurysm and dissection have been reported. Perugino et al. reported that among 160 patients with IgG4-RD, 11 (7%) had thoracic or AAAs, with two requiring surgical intervention for thoracic aortic dissection (15). Similarly, Hourai et al. identified IgG4-positive plasma cell infiltrates in 9.7% of various cardiovascular surgical specimens, particularly in the walls of dissecting thoracic aortic aneurysms (88). The vascular patterns and common complications of IgG4-related PAO/PA are depicted in **Figure 2**.

### 5.5 Comparison of IgG4-RD patients with/without PAO/PA

Patients with IgG4-related PAO/PA display distinct characteristics in demographics, organ involvement, inflammatory markers, and serum levels of IgG4 and IgE. Compared to patients with IgG4-RD who do not exhibit vascular involvement, those with IgG4-related PAO/PA are generally older at disease onset, have a higher prevalence of males, and experience a shorter disease duration. IgG4-related PAO/PA is often associated with highly active disease states and shows a more significant proportion of single-organ involvement. Conversely, involvement of the submandibular gland, lacrimal gland, and paranasal sinuses is less common. Compared to patients with IgG4-RD who lack periaortitis or periarteritis, those with IgG4-related PAO/PA typically present with higher white blood cell counts, erythrocyte sedimentation rate (ESR), and high-sensitivity CRP levels but have lower levels of blood hemoglobin, serum IgG4, and IgE (20). Regarding treatment, the effectiveness of steroid medications and the relapse rate during or after treatment do not significantly differ from other types of IgG4-RD (86).

## 6 Diagnostics and differential diagnosis of IgG4-related PAO/PA

### 6.1 Diagnostic criteria of IgG4-related PAO/PA

The diagnosis of IgG4-related PAO/PA requires an integrated approach that incorporates clinical, serological, radiological, and histopathological features. Current diagnostic frameworks include the 2011 CDC, the 2019 AECC, and the 2020 RCD (89–91). While the CDC and AECC emphasize vascular involvement in IgG4-related RF via arterial wall thickening on imaging, they may overlook isolated aortic or branch vessel lesions (90, 91). Furthermore, recent studies suggest that the 2019 AECC exhibits lower sensitivity for specific IgG4-RD phenotypes, particularly those involving the retroperitoneum and aorta, potentially leading to underdiagnosis in these subgroups (35, 36, 91). The 2020 RCD addresses this limitation by incorporating organ-specific criteria for PAO/PA and RF, thereby enhancing sensitivity for vascular involvement (72, 89). However, the RCD still demonstrates suboptimal performance for the “Retroperitoneum and Aorta” group, with only 66.7% of patients meeting the criteria (35). This underscores the persistent need for further refinement of diagnostic criteria to improve the identification of vascular involvement in IgG4-RD.

**Figure 3:**
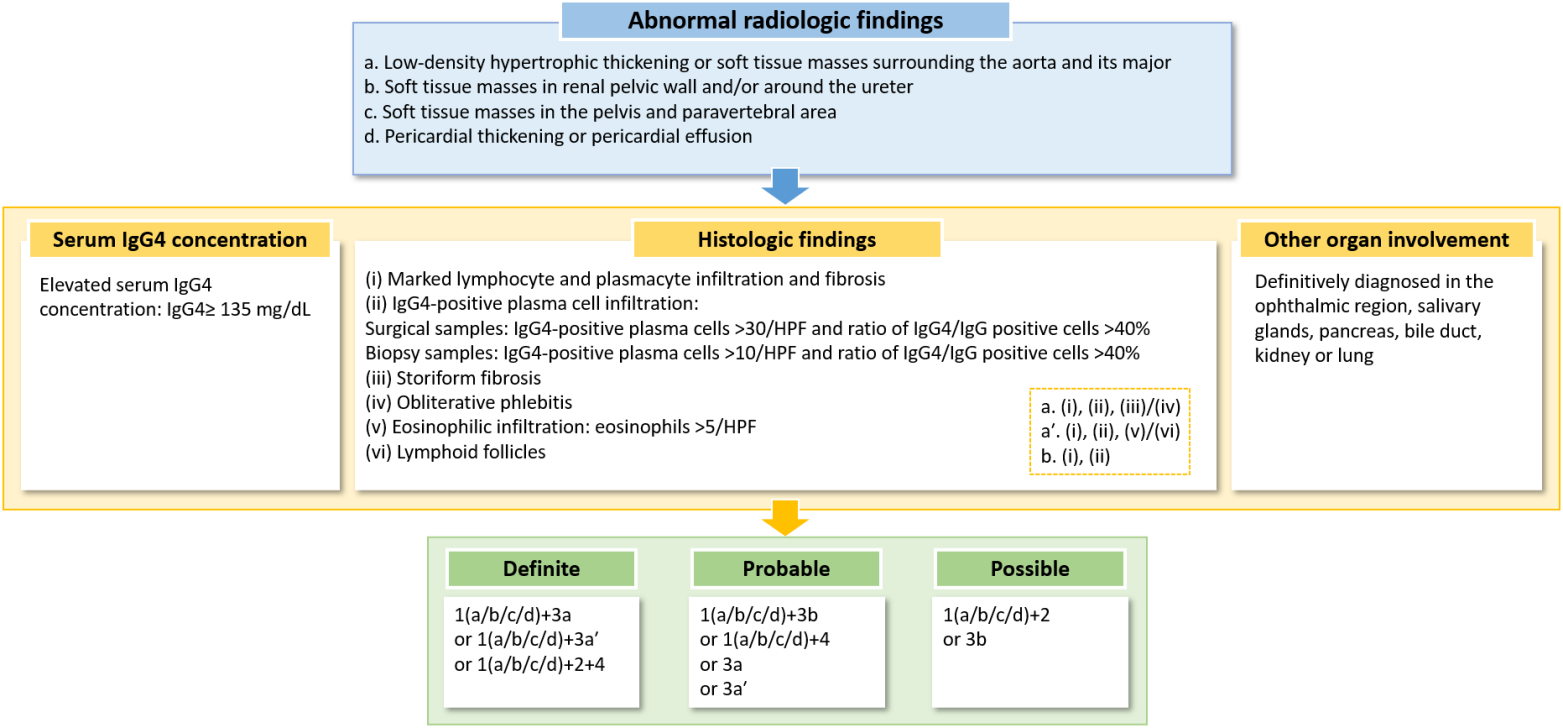
A diagnostic algorithm for IgG4-related cardiovascular/retroperitoneal disease. HPF, high-power field. Note, “/” indicates “or”.

A significant advancement is the 2018 organ-specific criteria (updated in 2023) proposed by Mizushima et al., which categorize IgG4-related PAO/PA as “definitive,” “probable,” or “possible” based on combined radiological, serological, and pathological findings (37). Crucially, these criteria permit diagnosis based solely on histopathology, independent of serum IgG4 levels—a critical adaptation given that IgG4 levels are not consistently elevated in histopathologically confirmed cases (92, 93). The 2023 revision further refines diagnostic accuracy for cardiovascular/retroperitoneal involvement, demonstrating 77.2% sensitivity and 94.7% specificity, though validation through larger multicenter studies remains essential (63). A diagnostic algorithm for IgG4-related cardiovascular/ retroperitoneal disease is illustrated in **Figure 3** (63).

### 6.2 Diagnostic examinations of IgG4-related PAO/PA

#### 6.2.1 Radiological imaging

Radiologic and histologic findings are essential for a “definitive” or “probable” diagnosis of IgG4-related PAO/PA. A “possible” diagnosis may be established based on radiologic findings combined with elevated serum IgG4 levels or histologic findings alone (37).

##### 6.2.1.1 CT features

CT imaging reveals concentric thickening of the aortic or arterial wall as a hallmark feature of IgG4-related PAO/PA. This thickening is typically smooth and symmetric, appearing hyperdense when enhanced with contrast. The underlying cause of this thickening is the dense infiltration of inflammatory cells and fibrosis within the adventitial layer of the aorta. IgG4-related PAO/PA may manifest as a soft tissue mass encircling the aorta or affected artery in certain instances. This mass is generally homogeneous and exhibits moderate enhancement following contrast administration, indicating significant inflammation and fibrosis surrounding the vessel (18).

**Figure 4:**
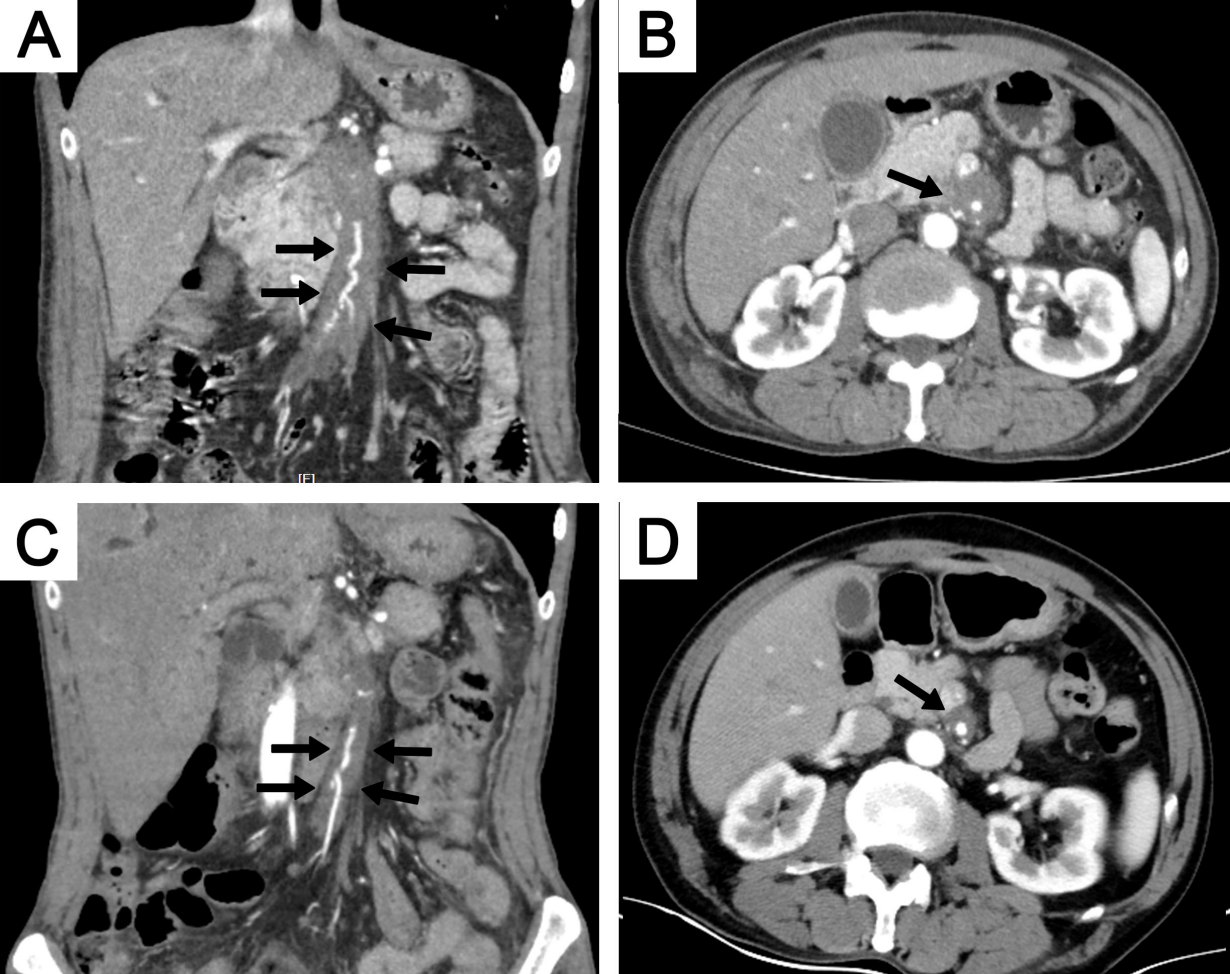
A 64-year-old man with involvement of the superior mesenteric artery. CECT showed soft tissue surrounding the superior mesenteric artery in both coronal **(A)** and axial **(B)** views (black arrows). The extent of the soft tissue decreased after 6 months of steroid treatment, as shown on coronal **(C)** and axial **(D)** views (black arrows). CECT, contrast-enhanced computed tomography.

CT imaging is highly effective in detecting aneurysms, luminal narrowing, or occlusion. However, severe aortic stenosis, which can occur in conditions like TA, has not been documented in patients with IgG4-related aortic lesions (37, 94). Luminal stenosis may be observed in medium-sized arteries, such as the coronary, internal carotid, or inferior mesenteric arteries (15, 83). While evaluating vessel wall thickness through CECT is a helpful indicator of vascular inflammation, it is crucial to recognize that increased wall thickness can persist long after the acute phase of arterial inflammation, potentially limiting its effectiveness in early assessments. Additionally, CT imaging cannot distinguish between active disease and fibrosis, further complicating the evaluation process (81). A representative case of involvement of the superior mesenteric artery is illustrated in **Figure 4**.

##### 6.2.1.2 Positron emission tomography/computed tomography

Given the limitations of CT in evaluating inflammatory activity, PET-CT serves as a critical adjunct for assessing disease activity and multi-organ involvement in IgG4-related PAO/PA (95–101). A previous study found that 40% of patients with IgG4-RD exhibited vascular involvement when assessed with FDG-PET/CT and CECT (26). PET scans usually show increased FDG uptake in inflamed areas of the aorta or other affected arteries, indicating active inflammation. Additionally, FDG-PET is useful for evaluating inflammation levels and guiding the selection of biopsy sites (102, 103). In a retrospective study assessing the effectiveness of PET and conventional imaging methods (ultrasound, CT, magnetic resonance imaging) in IgG4-RD, it was found that PET was more sensitive in detecting vascular lesions, including the aorta, large arteries, and other more accessible organs for biopsy (95). Yabusaki et al. reported that IgG4-aortitis-positive regions exhibited more than 2-fold the FDG uptake of the background blood pool (26). However, FDG uptake is non-specific in differentiating between atherosclerotic changes and active inflammation (104, 105).

To enhance the specificity of PET and assist in differentiating it from atherosclerosis, the authors proposed calculating the ratio of the arterial standardized uptake value max to that of the venous blood pool, known as the target-to-background ratio (26). Furthermore, PET imaging is crucial in monitoring treatment responses, demonstrating a strong correlation with therapeutic outcomes (95). A reduction in FDG uptake post-treatment indicates diminished inflammatory activity, while sustained or increased uptake may suggest refractory disease or a potential relapse (106–108).

#### 6.2.2 Laboratory tests

Laboratory investigations are crucial in diagnosing and managing IgG4-related PAO/PA. A combination of elevated serum IgG4 levels, inflammatory markers, peripheral eosinophilia, and hypocomplementemia can strongly indicate the presence of IgG4-related PAO/PA (22).

Elevated serum IgG4 levels are a hallmark laboratory finding in IgG4-related PAO/PA, but the diagnostic value and the specific cut-off level of serum IgG4 levels remain unclear (31, 109, 110). Wallace et al. discovered that only 51% of patients with biopsy-confirmed active IgG4-RD had elevated serum IgG4 levels. In their study, patients with elevated IgG4 levels were generally older, exhibited a higher IgG4-RD responder index, involved more organs, had lower complement levels, and showed higher eosinophil and IgE levels than those with normal IgG4 levels (31). Mizushima et al. reported that 92.5% of patients with IgG4-related PAO/PA exhibited increased serum IgG4 levels, and 77.5% showed elevated serum IgG levels (22). However, other studies indicated that IgG4 levels are not consistently elevated in IgG4-related PAO/PA or RF diagnosed histopathologically (92, 93). A recent study found that serum IgG4 levels were within the normal range in 71% of patients with IgG4-AAA diagnosed on surgical samples. Therefore, an elevated IgG4 serum level is not a reliable parameter for diagnosing IgG4-RD (69).

Inflammatory markers such as CRP and ESR help assess IgG4-RD activity and evaluate treatment response (20). In a Chinese cohort, ESR, high-sensitivity CRP, and IgA were higher in IgG4-related PAO/PA patients, but serum IgG4 and IgE levels were lower than those of IgG4-RD without vascular involvement (20). Several other studies also observed similar results, with CRP and ESR typically elevated in patients with IgG4-related PAO/PA compared to non-vascular IgG4-RD, the latter often showing normal serum CRP levels (47, 111–113). However, in the study by Ebe H et al., serum CRP levels were not necessarily elevated in patients with IgG4-related perivascular lesions, as compared to other vasculitis syndromes such as TA and GCA (74). The variation in inflammatory markers reported across studies may be attributed to the activity level of IgG4-RD and the number of organs involved. An elevated IgG4-RD activity state could also lead to periaortitis or periarteritis.

### 6.3 Differential diagnosis of IgG4-related PAO/PA

Distinguishing IgG4-related PAO/PA from conditions with overlapping vascular manifestations requires a systematic evaluation integrating clinical, histopathological, radiological, and serological features. Key differential diagnoses include large-vessel vasculitides such as TA and GCA, connective tissue diseases, other vasculitides like eosinophilic granulomatosis with polyangiitis (EGPA), and non-immune conditions including atherosclerosis, infectious aortitis, and malignancies (22, 107, 114).

TA usually affects younger Asian females and often presents with arterial stenosis, occlusion, diminished pulses, limb claudication, and inter-limb blood pressure differences. These clinical manifestations are less common in IgG4-related PAO/PA (115, 116). Histologically, TA is characterized by granulomatous inflammation with giant cells and necrosis. On imaging, aortic wall thickening in TA is often irregular and asymmetrical, commonly associated with stenosis and occlusion of the aorta and its branches, in contrast to the more concentric thickening seen in IgG4-related PAO/PA, which may instead lead to aneurysmal dilation. Additionally, pulmonary artery involvement is a distinctive feature of TA, but it is rare in IgG4-related PAO/PA (117).

GCA, the most common large-vessel vasculitis in older adults, also involves the aorta and its branches and can mimic IgG4-related PAO/PA (118). Key distinguishing features of GCA include its frequent association with systemic symptoms, such as headache, jaw claudication, polymyalgia rheumatica, and visual disturbances resulting from temporal artery involvement (119). While both conditions may show elevated ESR and CRP, GCA typically exhibits significantly higher systemic inflammation levels. Histologically, GCA is defined by granulomatous inflammation with multinucleated giant cells. Imaging often reveals irregular, patchier vascular wall thickening compared to the smoother, concentric pattern characteristic of IgG4-related PAO/PA. GCA also commonly affects the extracranial branches of the carotid arteries, whereas IgG4-related PAO/PA more frequently involves the aorta and its major abdominal branches (120).

Connective tissue diseases, such as rheumatoid arthritis, systemic lupus erythematosus, and primary Sjögren syndrome (pSS), should also be considered in the differential diagnosis. pSS, in particular, shares some clinical features with IgG4-RD, such as salivary and lacrimal gland enlargement causing sicca symptoms, lymphadenopathy, and an increased (though significantly higher in pSS) risk of lymphoma. However, pSS demonstrates a strong female predominance, characteristic autoantibodies (anti-Ro/SSA, anti-La/SSB, antinuclear antibodies, and rheumatoid factor), and polyclonal hypergammaglobulinemia, typically involving IgG1–3 subclasses, with serum IgG4 levels usually being normal or decreased (121). In contrast, IgG4-RD exhibits a male predominance, elevated serum IgG4 levels, and distinctive histopathological findings. Notably, isolated submandibular gland involvement (e.g., Küttner’s tumor) without parotid involvement is common in IgG4-RD but unusual in pSS (122–124).

Other vasculitides presenting diagnostic challenges include EGPA, Behçet’s disease, Cogan syndrome, and Kawasaki disease. EGPA shares features with IgG4-RD, including allergies, eosinophilia, elevated serum IgE, and even elevated serum IgG4 levels and tissue infiltration by IgG4-positive plasma cells (24, 125–127). Kubo et al. reported that over 60% of EGPA patients met the histopathological criteria for IgG4-RD, characterized by an IgG4-positive/IgG-positive plasma cell ratio exceeding 40% and >10 IgG4-positive plasma cells per HPF (128). However, EGPA typically manifests with asthma, peripheral neuropathy, and skin involvement, whereas IgG4-RD more commonly presents with mass-forming lesions in organs such as the pancreas or retroperitoneum. Crucially, histopathological hallmarks particular to IgG4-RD, such as storiform fibrosis and obliterative phlebitis, are key discriminators from EGPA (128).

Non-immune conditions such as atherosclerosis, infectious aortitis, and malignancies should be excluded. Atherosclerosis is frequently misdiagnosed as IgG4-related PAO/PA, especially in older patients, as both conditions can cause similar vascular changes. Careful identification of arterial wall calcification on imaging is a valuable clue pointing towards atherosclerosis rather than IgG4-related inflammation (129). **Table 3** summarizes the key differential diagnoses of IgG4-related PAO/PA, TA, GCA, and atherosclerosis.

**Table 3:** Differential diagnosis of IgG4-related PAO/PA.

Feature	IgG4-related PAO/PA	Takayasu Arteritis	Giant Cell Arteritis	Atherosclerosis
Sex	Male>female	Male<female	Male<female	Male≈female
Age	>60 years	20–30 years	>50 years	>60 years
Common site	Abdominal aorta	Thoracic aorta	Temporal artery, thoracic aorta	Abdominal aorta
IgG4/CRP/ER	IgG4↑↑↑/CRP↑/ESR↑	CRP↑↑↑/ESR↑↑↑	CRP↑↑↑/ESR↑↑↑	Normal
Image	Smooth, concentric vascular thickening	Irregular and asymmetrical vascular thickening	More irregular and patchier thickening	Calcification of the arterial wall
Histopathology	Lymphoplasmacytic infiltration, IgG4-positive plasma cells, storiform fibrosis, and obliterative phlebitis in the adventitia layer	Granulomatous inflammation with giant cells and necrosis in both adventitia and media layer	Multinucleated giant cells and granulomatous inflammation in the media layer	Lipid-rich plaques, foam cells, and calcification in the intimal layer
Other organ involvement	Pancreas, kidney, retroperitoneal fibrosis, etc.	No	No	No
Complications	Most common aortic Aneurysms; others include aortic dissections, vascular stenosis, occlusion, and compression.	Vascular stenosis and occlusion	Vascular stenosis and occlusion	Vascular stenosis and occlusion

IgG4-related PAO/PA, IgG4-related aortitis/periaortitis and periarteritis; CRP, C-reactive protein; ESR erythrocyte sedimentation rate.

## 7 Treatment and prognosis of IgG4-related PAO/PA

### 7.1 Drug treatment

GC therapy is the first-line treatment for IgG4-RD. Early initiation of corticosteroid therapy can prevent irreversible damage, such as fibrosis, and reduce the risk of vascular complications, including aneurysm formation (130). The typical starting dose of prednisone is 30–40 mg/day (approximately 0.6–1.0 mg/kg/day), adjusted according to disease severity. Patients generally experience a rapid reduction in symptoms and inflammatory markers, such as CRP and ESR, within 2–4 weeks of starting treatment. Once clinical improvement is achieved, the prednisone dose is gradually tapered over 3–6 months. Maintenance therapy with a relatively slow taper to 5–10 mg/day by 12 months may show good efficacy (22). A previous systematic review, including six studies, reported that all studies showed a good response to treatment, primarily with corticosteroids (21). Radiographic improvement of more than 50% reduction in thickness was observed 2 months after therapy, and 53% of cases had almost complete resolution (22). However, for asymptomatic IgG4-related PAO/PA without organ damage, corticosteroid therapy should be used with caution.

Steroid-sparing agents have not been well-established in IgG4-RD. However, IMs, including azathioprine, methotrexate, and mycophenolate mofetil, have been reported to be effective in case reports and case series. Over 90% of patients with IgG4-related PAO/PA treated with GCs in combination with IMs achieved complete remission (20). Although IMs are less effective than corticosteroids in inducing remission, they are essential for maintaining long-term disease control, especially in steroid-dependent patients or those with contraindications to prolonged corticosteroid use (131).

Rituximab, a monoclonal antibody targeting CD20-positive B cells, has been used in both steroid-refractory cases and as initial therapy (as monotherapy or in combination with steroids) and has demonstrated high efficacy in reducing inflammation and controlling disease activity in patients with IgG4-RD, including those with vascular involvement (15, 132–135). It depletes B cells, which are key contributors to the immune dysregulation seen in IgG4-RD, by inhibiting the production of IgG4-positive plasma cells. Clinical trials have shown that rituximab (NCT01584388) significantly reduces serum IgG4 levels, improves imaging findings, and reduces relapse rates (132). However, its efficacy and influence on luminal dilatation in IgG4-related PAO/PA remain to be evaluated.

IL-6 is a pro-inflammatory cytokine that plays a crucial role in the pathogenesis of many chronic inflammatory diseases, including IgG4-RD. Elevated levels of IL-6 have been identified in patients with IgG4-RD, correlating with disease activity (136–138). Tocilizumab, a monoclonal antibody against the IL-6 receptor, has been reported to successfully treat patients with IgG4-RD, either as monotherapy or in cases refractory to steroids, rituximab, and azathioprine (139–141). A prospective cohort study highlighted the positive response to tocilizumab in a fixed 6-month treatment regimen among 14 patients, both treatment-naïve and those refractory to other therapies, achieving a 50% complete response rate (142). A recent report also detailed the successful treatment of two patients with steroid-refractory IgG4-related aortitis and RF using tocilizumab (143). Although limited data specifically address the use of IL-6 inhibitors in IgG4-related PAO/PA, tocilizumab offers a promising therapeutic option for patients with IgG4-related PAO/PA, especially those who are refractory to standard treatments or cannot tolerate GCs.

Emerging B cell-targeting agents such as inebilizumab and obexelimab show promise in treating IgG4-RD. Inebilizumab, a B-cell-depleting anti-CD19 monoclonal antibody, reduced the risk of IgG4-RD flare by 87% compared with placebo over 52 weeks in a phase III randomized placebo-controlled trial (144). Obexelimab, which coligates CD19 and FcγRIIb to inhibit B cells without depletion, is currently under investigation in a phase III trial (145). T-cell-targeting agents such as abatacept, a synthetic analogue of cytotoxic T lymphocyte antigen 4, have demonstrated efficacy in some patients with active IgG4-RD (146). Janus kinase (JAK) inhibitors represent another promising alternative, with tofacitinib proving effective in inducing responses in patients with IgG4-RD and idiopathic RF (147). Ongoing trials are investigating the efficacy of JAK inhibitors, including tofacitinib (NCT05625581) and baricitinib (NCT05781516), in treating IgG4-RD.

### 7.2 Surgical intervention

Surgical intervention is required in patients with AAAs to prevent rupture, and repair is typically indicated when the aortic diameter exceeds 5.5 cm. Previous studies have suggested that endovascular repair is associated with lower inflammatory process resolution rates than open surgery (142, 148, 149). Open surgery is technically challenging because inflammatory AAAs are often associated with dense adhesions surrounding the aneurysm (150). For example, Kasashima et al. reported a case of IgG4-related inflammatory AAA where the patient died of duodenal rupture and acute peritonitis on the third postoperative day, likely due to tight fibrous adhesion between the abdominal aorta and the duodenum (58).

Surgical procedures have not been well-documented in patients with IgG4-related PAO/PA. Emergency surgical repair may be required in cases of rapid aneurysm enlargement (151). Perugino et al. performed vascular surgeries on seven patients with primary IgG4-related vasculitis, including carotid endarterectomy, coronary artery bypass grafting, and open aortic aneurysm repair. All procedures successfully addressed the underlying vascular issues, and each surgical patient received rituximab postoperatively (15). Notably, patients who received rituximab without surgery exhibited stable or improved vascular findings on follow-up imaging, highlighting the importance of early detection and proactive medical therapy in preventing surgery and adverse outcomes (15). Another study involving patients with chronic periaortitis, including IgG4-related PAO/PA, showed that those who underwent surgical or endovascular repairs maintained sustained patency without recurrence of aneurysms (16). However, it is essential to recognize that surgery does not resolve the underlying immune-mediated inflammation. Therefore, ongoing medical management with immunosuppressive therapy is necessary to prevent further vascular damage (152, 153).

### 7.3 Prognosis of IgG4-related PAO/PA

Most IgG4-related PAO/PA patients respond well to medical or surgical treatment. However, careful monitoring of aneurysms and dissections is essential during follow-up. Several studies have reported that patients with pre-treatment aneurysms are at higher risk of aneurysm expansion and progression after corticosteroid treatment, occurring in 20.9% to 50% of cases. This risk is attributed to corticosteroids potentially weakening the aneurysm wall, thereby increasing the likelihood of rupture in patients with pre-existing aneurysms (17, 18, 22, 85, 154). High-dose corticosteroid therapy may further exacerbate this risk by thinning the arterial wall (155). However, spontaneous rupture of aortic aneurysms has also been observed in IgG4-related PAO/PA patients who did not receive corticosteroid treatment (156, 157).

Inoue et al. suggested that administering a lower dose of corticosteroids, precisely 20 mg daily, may reduce the risk of aneurysmal rupture. This dosage effectively reduces aortic wall thickening and swelling in the pancreas and bile duct but does not significantly alter the aneurysmal diameter during therapy (18). Similarly, Peng et al. treated nine patients with aneurysmal dilation using a moderate dose of GCs (0.5 mg/kg) combined with immunosuppressive therapy. No significant dilation extent or diameter changes were observed, and none of the patients experienced aneurysmal rupture (20). Additional studies have reported no significant alterations in aneurysm diameters or dilation following corticosteroid treatment (22, 75). While it remains unclear whether corticosteroid treatment accelerates aneurysm progression, according to limited studies, a low-dose steroid strategy (no more than 20 mg/day or [0.6 mg/kg/day]) is considered safe. However, it is crucial to closely monitor any pre-existing luminal dilation before initiating corticosteroid therapy (17).

The relapse rate of IgG4-related PAO/PA has been documented in a limited number of studies. A survey by Kasashima et al. reported relapse in approximately 20% (6/31) of patients with IgG4-related PAO/PA (median follow-up 47 months) and other organ involvement (median follow-up 56 months) after achieving initial remission with corticosteroid therapy (86). In contrast, Peng et al. observed a significantly lower relapse rate of 5.6% (5/89) during a median follow-up of 21 months among patients treated with combined GCs and IMs. This suggests that combination therapy may be more effective in preventing relapse than corticosteroids alone. Furthermore, all relapses in their study occurred in patients involving two or more organs at baseline, with recurrence observed in non-vascular sites (20).

A recent multicenter, open-label, randomized controlled trial from China investigated a treatment withdrawal strategy in patients with long-term stable IgG4-RD receiving low-dose GCs combined with IM maintenance therapy. Within an 18-month follow-up period, disease relapse occurred in 25 of 48 patients (52.1%) who withdrew both GCs and IMs. In contrast, the relapse rate was significantly lower in patients who withdrew GCs but maintained IMs (7/49, 14.2%) and those who continued both therapies (6/49, 12.2%). This study provides strong evidence that maintaining IMs, with or without low-dose GCs, is superior to withdrawing them in preventing relapse in IgG4-RD (158). The treatment and outcomes of IgG4-related PAO/PA are listed in **Table 4**.

**Table 4:** Treatment and outcomes of IgG4-related PAO/PA.

Author(year)	Observation period	Treatment	Outcomes	Ref.
Inoue et al. (2011)	9 months (median)	PSL, surgery	10 patients showed improvement in wall thickening. Among them, 1 patient exhibited spontaneous reduction in wall thickening during follow-up without any treatment. The luminal diameters of the affected vessels remained unchanged in all lesions, including the aneurysmal aorta in 1 case after steroid therapy.	(18)
Mizushima et al. (2014)	30 months (mean)	PSL, CTX	30 patients showed improvement in wall thickening. However, 1 patient experienced a relapse during the tapering of PSL. 2 patients exhibited exacerbations, both of whom had undergone luminal dilation before therapy.	(22)
Ebe et al. (2015)	3–6 months	PSL	7 patients showed a decrease in serum IgG4 levels; among them, 6 showed improvement in wall thickening.	(74)
Perugino et al. (2016)	NS	Rituximab, PSL, rituximab plus PSL, tamoxifen plus prednisone, surgery	Of the 13 patients treated with rituximab, 9 showed clinical improvement, 5 showed radiologic improvement, 5 remained unchanged, and 1 experienced worsening. All surgeries were successful.	(15)
Ozawa et al. (2017)	NS	PSL	43 patients showed improvement of wall thickening, but 9 patients exhibited worsening of luminal dilatation.	(17)
Kim et al. (2017)	Over 6 months	PSL, AZA, MTX, MMF, surgery	7 patients showed remission, but 3 patients showed persistent activity.	(16)
Qi et al. (2019)	10 months (median)	PSL	13 patients showed improvement in wall thickening, but 2 patients exhibited slight enlargement of vascular diameter.	(75)
Peng et al. (2020)	6 months	PSL, CTX, MMF, leflunomide, tamoxifen	34 patients achieved a reduction in perivascular soft tissues of more than 70%, 39 patients achieved a reduction between 31% and 70%, and 16 patients had a reduction of less than 30%. Additionally, 5 patients relapsed.	(20)

IgG4-related PAO/PA, IgG4-related aortitis/periaortitis and periarteritis; PSL, prednisolone; CTX, cyclophosphamide; AZA, azathioprine; MTX, methotrexate; MMF, mycophenolate; NS, not specified.

## 8 Conclusion

IgG4-related PAO/PA is a complex disorder requiring integrated diagnostic approaches. While its clinical manifestations resemble those of other IgG4-RD phenotypes, the distinct risks of aneurysm rupture and hydronephrosis necessitate heightened vigilance. Current diagnostic criteria remain imperfect and need further refinement through research. GCs are effective as first-line therapy, but long-term use requires balancing efficacy with adverse effects. Emerging B- and T-cell-targeted therapies offer promise; however, significant unmet needs persist in early detection, risk stratification, and complication prevention. Given the disease’s rarity, multicenter collaborative studies with extended follow-up are imperative to advance our understanding of its pathogenesis, optimize therapeutic strategies, and improve outcomes.
